# Modified Clover Technique Using Automated Suture Placement and Securing Technology in a Passive Beating Heart Model

**DOI:** 10.3390/bioengineering11070666

**Published:** 2024-06-29

**Authors:** Severin Laengle, Aldo Suria, Thomas Poschner, Sahra Tasdelen, Antonios Pitsis, Alfred Kocher, Martin Andreas

**Affiliations:** 1 Department for Cardiac Surgery, Medical University of Vienna, 1090 Vienna, Austria; aldo.suriaroldan@meduniwien.ac.at (A.S.);; 2 European Interbalkan Medical Center, Thessaloniki Heart Institute, 57001 Thessaloniki, Greece

**Keywords:** tricuspid regurgitation, clover technique, edge-to-edge repair

## Abstract

Tricuspid regurgitation (TR) is a prevalent finding in echocardiography and in case of severe disease is associated with impaired patient outcome. Clover repair offers a surgical solution that can be applied for the treatment of primary and secondary TR. An ex vivo passive beating porcine heart model was created to test a modified clover technique using automated suturing devices and to compare this approach to standard ring annuloplasty. Secondary TR was induced in 10 porcine hearts and the backflow of fluid was assessed. The primary endpoint of this study was regurgitant volume measured in mL at the site right atrial cannula. The baseline regurgitation was 43.3 ± 10.8 mL. The mean regurgitant volume was significantly reduced after all repair procedures to 22.2 ± 5.9 mL with isolated ring annuloplasty, 12 ± 3.9 mL with the modified clover, and 7.6 ± 3.4 mL with the combined procedure (*p* < 0.0001). The modified clover technique shows how to effectively reduce TR in an ex vivo model. This method may be suitable to facilitate tricuspid repair, especially for totally endoscopic valve surgery.

## 1. Introduction

The incidence of at least moderate tricuspid valve regurgitation (TR) reported from epidemiologic studies is estimated between 5 and 8% [1,2]. Patients suffering from severe TR have a reduced overall survival [3]. Based on the morphology and etiology of the disease, the current classification distinguishes between primary, secondary, and cardiovascular implantable electronic device (CIED)-related TR [4,5]. Secondary tricuspid regurgitation (STR) can be caused by atrial and ventricular mechanisms depending on the cause of illness [6]. Most often, severe TR is related to secondary causes, for example, mitral valve disease and pulmonary hypertension [7,8]. Atrial-type secondary TR represents a distinct disease entity that is, among other features, marked by a disproportional enlarged right atrium compared to the right ventricle with concomitant annular dilatation [9]. Systolic flow reversal in the hepatic veins, a vena contracta ≥0.7 cm^2^, or a regurgitant volume ≥45 mL pre-beat are indicative of severe TR [10,11]. The decision for intervention should be based on the underlying disease etiology and the comorbidities of the patient. Surgical correction is recommended for symptomatic patients with isolated severe primary TR and in case of severe TR when left-sided valve surgery is performed [12]. In general, tricuspid valve repair (TVr) should be preferred over valve replacement [13]. Further, beating heart repair seems to offer benefits for patient outcome [14]. Surgical results after isolated tricuspid surgery improved in the last decade due to improved risk assessment and perioperative management, but the surgical approach for repair itself may also offer benefits for short- and long-term outcomes [15,16]. Several techniques have been described but most often, TVr is performed through ring annuloplasty [17]. The clover technique as an alternative approach has first been described to treat cases of primary, post-traumatic TR [18]. Since then, this method has been used in mixed patient populations with recurrent rates that are more than a mild TR of 17% at 10 years [19]. A combined procedure of annuloplasty and clover can also be performed, but only limited evidence on the efficacy of this approach is currently available. However, a combined approach may add beneficial effects of leaflet coaptation and annular stabilization. Importantly, annuloplasty sizing has to be modified to a larger ring if an additional clover technique is performed to improve the geometry and avoid transvalvular gradients.

Minimal invasive approaches for tricuspid valve surgery using a lateral thoracotomy with endoscopic visualization have emerged over the last decades and have shown favorable outcomes in the case of isolated and concomitant procedures [20,21,22]. The present study evaluated a modified procedure for approximating tricuspid leaflets using automated technology for suture placement and securing in an ex vivo porcine passive beating heart model. This technique may be especially useful in microinvasive procedures, in which there is reduced working space, and obtaining adequate visualization can be difficult.

## 2. Material and Methods

An ad hoc TR test bench was designed as a porcine passive beating heart model to show feasibility and evaluate the efficacy of repair procedures. A mock loop was created using a pulse duplicator to generate circulation inside the system. Different flow and pressure conditions can be generated to simulate basic valvular functions.

### 2.1. Passive Beating Heart Model

**Figure 1:**
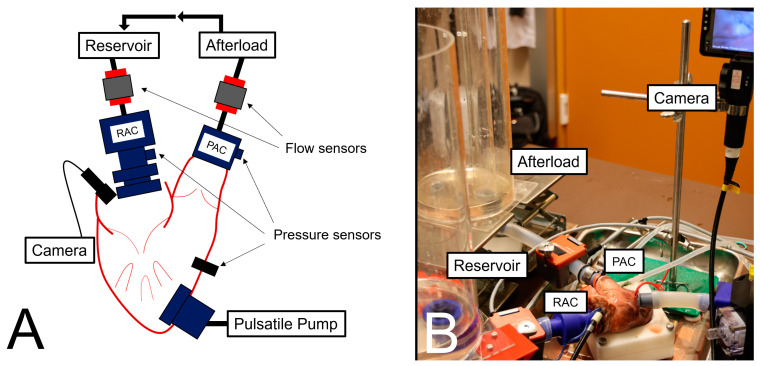
(**A**) Schematic representation of the passive beating heart model. (**B**) Photo of an ex vivo pulsatile setup with porcine heart.

The test bench used in this study permitted the attachment of a pulsatile pump to the right ventricle and to add preload and afterload to the heart. Three-dimensional-printed connectors were attached to the right atrium and the pulmonary artery. These were used for in- and outflow of working fluid (0.9% saline solution). Flow was provided by a pulsatile pump system (ViVitro Labs Inc., Victoria, BC, Canada), which was coupled to the right ventricle through a third connector. The forward and backward flow of working fluid were assessed using ultrasonic transit time measurement (SONOTEC GmbH, Halle, Sachsen-Anhalt, Germany) near the site of the right atrium connector (RAC) and pulmonary artery connector (PAC). In addition, three sensors (WIKA Alexander Wiegand SE & Co. KG, Klingenberg am Main, Germany) were used to record the pressure at the site of the RAC, the right ventricle, and the PAC. To directly check valve function during the experiments, a flexible camera was inserted through the right atrial appendage. The setup of the test bench is depicted in Figure 1. The pulse duplicator was set to generate stroke volumes between 67 and 76 mL with 50 beats per minute and 40% systole during one cycle. To induce TR, the annulus was mechanically dilated to 43 mm for 10 min before the experiment, and an increased afterload was created by a water collum after the PAC. Different sizes for mechanical dilatation were used in preliminary experiments. Experience showed this diameter worked best for small- and bigger-sized pig hearts.

### 2.2. Surgical Repair Techniques

**Figure 2:**
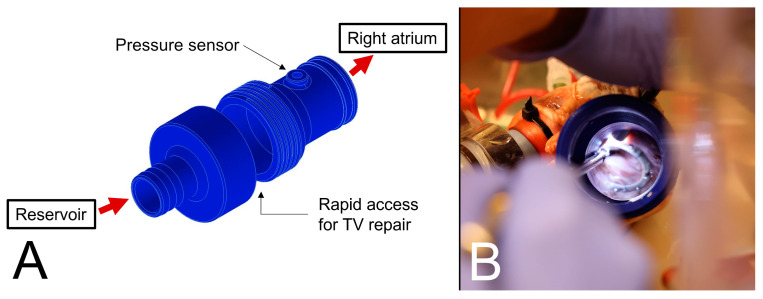
(**A**) A 3D CAD model of the right atrium connector. (**B**) Surgical implantation of the annuloplasty ring through the opened RAC.

The valve morphology of each specimen was assessed to ensure that no prolapse or leaflet perforation was present. After a baseline assessment of established regurgitation, surgical repair of the valve was achieved in three steps. First, annuloplasty was performed in a standard fashion using a Physio Tricuspid (Edwards Lifesciences, Irvine, CA, USA) or a Contour 3D (Medtronic plc, Dublin, Ireland) ring. Both 26 mm ring types were implanted in five hearts each. For the second step, a modified clover repair was carried out. Like the classic technique, all three leaflets of the tricuspid valve are plicated at the center of the valve. Modifications include the use of an automated suturing device (LSI SOLUTIONS, Rochester, NY, USA) to place horizontal mattress sutures through the leaflets instead of using one single suture. Mattress sutures with a pledget are placed through the middle of the free edges of the anterior, posterior, and septal leaflet. The second modification is the use of two automated titanium fasteners (LSI SOLUTIONS, Rochester, NY, USA) for tying the central knots. Three tails, one of each mattress suture, were tied with one fastener. The conduction of the modified clover repair is shown in Video S1. In the final step, the implanted annuloplasty rings were explanted, and measurements were taken for the isolated clover repair. The unscrewing of the RAC enabled rapid access to the tricuspid valve to perform all TVr techniques under direct vision (Figure 2). Results of the repair procedure can be seen in Figure S1 and Video S2.

### 2.3. Statistical Analysis

The results of our experiments were presented with means of descriptive statistics; continuous variables were expressed using mean and standard deviation. Categorical variables were presented as absolute numbers and percentages for each category. This study’s primary endpoint was the regurgitant volume, measured in mL at the inflow site (2–3 cm upstream of the RAC). The secondary endpoint of this study was the transvalvular pressure described as the mean pressure gradient (mPG) across the tricuspid valve in mmHg during diastole. For primary and secondary endpoint analysis, a two-way ANOVA using Tukey’s multiple comparison test with a level of significance of 0.05 was used. Sample size calculation was omitted due to the explorative and descriptive nature of our study. Statistical analysis was conducted using Microsoft Excel (Microsoft, Redmond, WA, USA), SPSS Statistics 27.0 (IBM, Armonk, NY, USA), and GraphPad Prism 8.0 (GraphPad Software, San Diego, CA, USA).

## 3. Results

**Figure 3:**
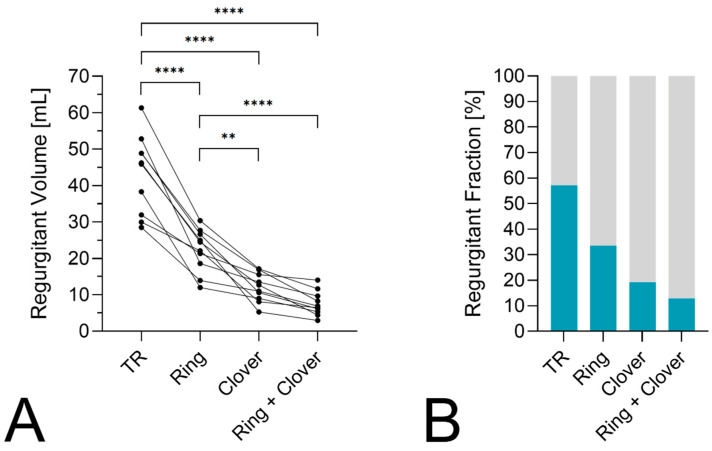
(**A**) Primary endpoint of regurgitant volume in mL shown for STR after ring annuloplasty, the clover procedure, and the combined repair. Regurgitation significantly decreased with all techniques compared to the baseline. Additionally, higher TR was present with the ring compared to the clover and the ring + clover. (**B**) The regurgitation fraction is illustrated for each condition with a reduction after the TVr procedures. ** *p* < 0.01, **** *p* < 0.0001.

Ten porcine hearts with a mean weight of 408 ± 48 g were used for the experiments. After connecting the heart to the pump, the loop was filled with working fluid, and baseline measurements were taken. The mean pulmonary artery pressure for initial regurgitation was 24 ± 3 mmHg. The mean regurgitant volume for TR was 43.3 ± 10.8 mL. TR decreased in all samples after annuloplasty. The mean regurgitant volume with rings was 22.2 ± 5.9 mL. The modified clover repair was successfully performed in all samples. The mean regurgitant volumes with clover and annuloplasty + clover alone were 12.0 ± 3.9 mL and 7.6 ± 3.4 mL, respectively (Figure 3). All three repair techniques significantly reduced TR compared to baseline conditions (*p* < 0.0001). The mean regurgitant volume was further lowered with the clover technique and the combined approach compared to ring annuloplasty alone (*p* = 0.0017 and *p* < 0.0001). The regurgitant fraction (RF) as the proportion of backflow measured at the RAC divided by the total volume measured during systole at the RAC and PAC was also lowered in all repair techniques. The mean RF for baseline TR, ring annuloplasty, clover repair, and the combined procedure were 57 ± 13%, 34 ± 9%, 19 ± 6%, and 13 ± 5%, respectively.

**Figure 4:**
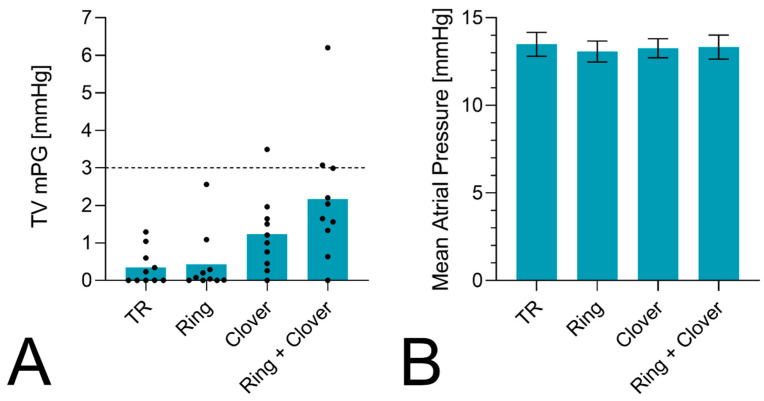
(**A**) The mean tricuspid valve pressure gradient after different procedures. A significant increase in the mPG was seen after the ring + clover compared to TR (*p* = 0.0025) and annuloplasty alone (*p* = 0.0039). (**B**) The mean atrial pressure in mmHg depicted for all conditions. No difference between the four experimental states was observed.

The mPG slightly increased after TVr from 0.4 ± 0.5 mmHg at the baseline to 0.4 ± 0.8, 1.2 ± 1, and 2.2 ± 1.7 mmHg after ring annuloplasty, clover repair, and the combined procedure (Figure 4). Multiple comparisons showed a significant difference in the mPG of the ring with clover compared to baseline TR (*p* = 0.0025) and annuloplasty (*p* = 0.0039). An mPG > 3 mmHg was observed in three hearts, one after performing a clover repair and two after a combined procedure. The mean atrial pressure did not differ between the four different conditions.

## 4. Discussion

Passive beating heart models for testing the clover technique using conventional or interventional methods have been described in the past [23,24]. Compared to our investigation, previous studies focused on the conventional technique without the use of mattress sutures and automated surgical devices. Amedi et al. performed an analysis of ring and focal suture annuloplasty in an ex vivo pulsatile flow loop and they showed a comparable reduction in TR [25]. In contrast, we describe a different leaflet plication technique and repeated the intervention on the same specimen through a rapid-access RAC. Furthermore, it should be mentioned that the use of mechanical annular dilatation, as well as an increased afterload with ventricular dilatation, makes our model more representative of mixed disease than only one type of secondary TR.

Annuloplasty has been the preferred treatment approach to TVr for several decades, with the implantation of rings showing a more favorable outcome than elastic bands [26,27]. The clover technique can be applied to treat primary TR, and CIED-related TR and has also been used for secondary TR with and without ring annuloplasty [19,28,29]. Moreover, a clover-like repair technique can even be performed in percutaneous edge-to-edge repair [30]. The clover technique as a versatile procedure shows satisfactory long-term durability. Most recent retrospective data show a rate of more than a mild TR of 23.8% at a follow-up of 16 years, adjusted for death as a competing risk factor [31]. It should be mentioned that 36% of patients received only suture annuloplasty or no annuloplasty at all. A previous trial showed an increased rate of pacemaker implantation after TVr with ring annuloplasty in patients with secondary TR after concomitant mitral surgery [32]. Using an isolated leaflet plication technique in cases of ventricular secondary TR may address TR and mitigate the risk of conduction disturbances with a subsequent need for pacemaker implantation.

Stenosis may arise after performing an edge-to-edge repair, especially in combination with annuloplasty, as seen in our experiments. Transvalvular gradients ≥ 5 mmHg indicate severe stenosis, which can lead to increased right atrial pressures and congestion [33]. It should be noted that no specific sizing protocol for the annuloplasty was used in this study. When performing a combined procedure though, the use of larger rings is preferred to mitigate the risk of stenosis [34]. We only advocate for the use of the modified clover technique if stenosis is avoided, although gradients of up to 4 mmHg can be acceptable in cases of residual TR.

The modifications described in our study could decrease complexity and broaden the application of this approach. Nowadays, TVr can be accomplished on a beating heart and through total endoscopic access [35,36]. The integration of automated knotting technology for triple edge-to-edge TVr can be especially useful during these procedures [37].

### Limitations

Several aspects should be considered before interpreting our results. Despite their widespread use in cardiovascular research, pig hearts have distinct features, most pronounced is the ventricular walls’ thickness, limiting the transfer of the results to the human body. Due to the nature of a passive beating heart model, physiologic contraction and the behavior of the right ventricle cannot be simulated. The expansion of the right ventricle, which can be observed on secondary TR, was present in our experiments, but the influence of increased afterload on right ventricular function and geometry was not considered in this study. Furthermore, possible effects of the left ventricle were not represented in our mock loop.

## 5. Conclusions

This new clover technique was demonstrated to successfully reduce TR in an ex vivo porcine heart model. Automated technology for suture placement and securing offers a promising approach, which may reduce the technical difficulty of performing the clover technique, including through microinvasive access.

## Data Availability

Raw data supporting the reported results are available upon request.

## Supplementary Materials

The following supporting information can be downloaded at https://www.mdpi.com/article/10.3390/bioengineering11070666/s1, Figure S1: Representative pictures of ring annuloplasty (A), clover repair (B) and the combined procedure (C) performed through the right atrial connector.; Video S1: Exemplary video showing the modified clover technique using automated suture placement and securing technology.; Video S2: Footage of a borescope camera inside the right atrium.

## Abbreviations

CIED	Cardiovascular implantable electronic device
mPG	Mean pressure gradient
PAC	Pulmonary artery connector
RAC	Right atrium connector
RF	Regurgitant fraction
TVr	Tricuspid valve repair
TR	Tricuspid regurgitation
