# Supplementary figures and images for: Modified Clover Technique Using Automated Suture Placement and Securing Technology in a Passive Beating Heart Model
Source: Bioengineering (Basel). 2024 Jun 29;11(7):666. doi: 10.3390/bioengineering11070666 (PMC11273994; doi:10.3390/bioengineering11070666)

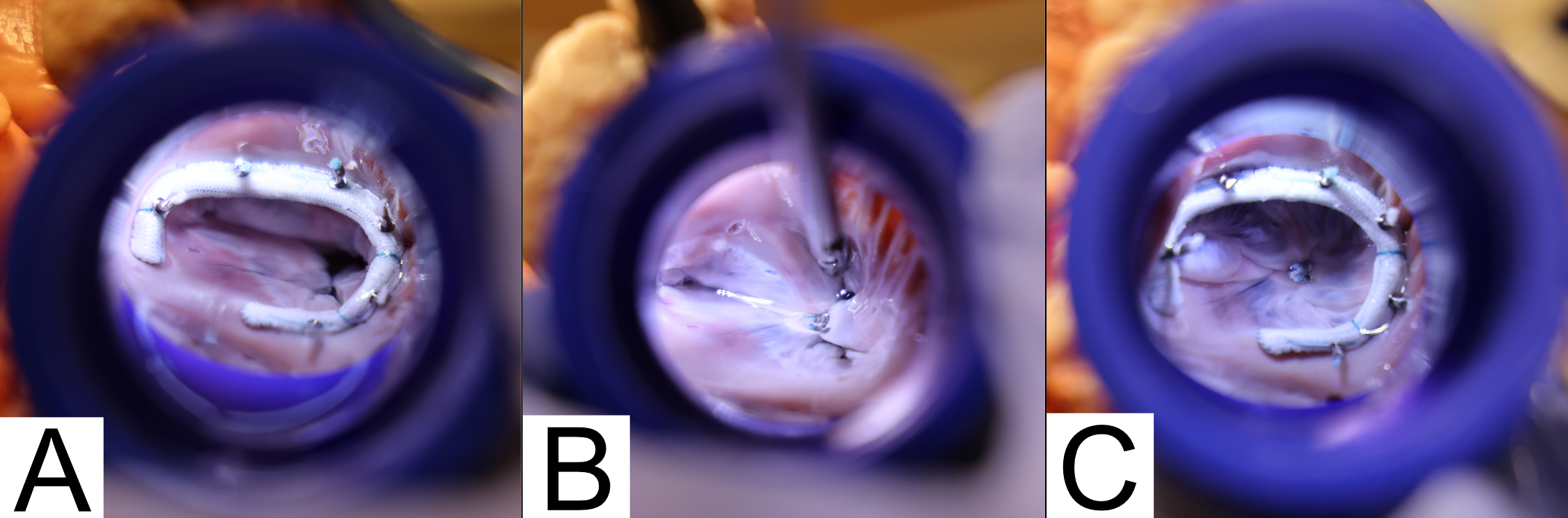

Supplement: Supplementary file 1 [file bioengineering-11-00666-s001.zip › bioengineering-3061823-supplementary-Figure S1.tif]
